# The complete mitochondrial genome of dark mealworm *Tenebrio obscurus* (Coleoptera: Tenebrionidae)

**DOI:** 10.1080/23802359.2019.1642155

**Published:** 2019-07-16

**Authors:** Yan-Hua Zhang, Yan-Yu Zhao

**Affiliations:** a School of Life Sciences, Jiangsu Normal University, Xuzhou, China;; b Department of medical genetics, Zunyi Medical University, Zunyi, China

**Keywords:** *Tenebrio obscurus*, mealworm, edible insect, mitogenome

## Abstract

Mealworms are the most common edible insect and feed additives in poultry, livestock, and aquaculture production. In the present study, the complete mitochondrial genome of dark mealworm *Tenebrio obscurus* (Zunyi population) was sequenced and annotated. The 15,509 bp circular mitogenome contains a conserved set of 37 mitochondrial genes including 13 protein-coding genes, 22 tRNA genes, 2 rRNA genes, and an A + T-rich region. The gene order is identical to the typical insect gene arrangement. Great differences were found in A + T-rich regions between Zunyi population and Guiyang population including large deletion and substitution. Phylogenetic analysis showed that *T. obscurus* clustered within Tenebrionoidea and was closely related to *Tenebrio molitor*.

Mealworms are the larvae of two species of darkling beetles, the yellow mealworm (*Tenebrio molitor*), and dark or mini mealworm (*Tenebrio obscurus*), which is regarded by global food system experts as potentially one of the most valuable food source for humans (Martin et al. 1976; Vantomme et al. 2012). Although mealworms are considered as the pest of stored grain in some countries, actually they have been widely used as the most common food and feed additives in poultry, livestock, and aquaculture production (Klasing et al. 2000; Ballitoc and Sun 2013). Recently the population structure and phylogenetic position of *T. molitor* have been investigated using mitochondrial data (Liu and Wang 2014; Zhang et al. 2016), while there is only one mitochondrial sequence of *T. obscurus* available from GenBank (Bai et al. 2018). In the present study, we report the mitogenome of *T. obscurus* (Zunyi population), which will provide valuable information for molecular phylogenetics and population genetics.

Specimens of *T. obscurus* (JNU20160522) were provided and preserved by the cell biology laboratory of Jiangsu Normal University. The complete *T. obscurus* mitogenome was amplified by using a combination of universal primers (Zhang et al. 2016) and annotated using MITOS web server (Bernt et al. 2013). The phylogenetic trees were reconstructed from 26 Cucujiformia mitogenomes using Bayesian inference (BI) and maximum-likelihood (ML) methods.

The complete mitogenome of *T. obscurus* (GenBank accession number: KY270783) is 15,509 bp in size, which is shorter than *T. obscurus* mitogenome of Guiyang population. It contains a conserved set of 37 mitochondrial genes including 13 protein-coding genes (PCGs), 22 tRNA, 2 rRNA genes, and an A + T-rich region. The gene order is identical to the typical insect gene arrangement. The heavy chain codes 23 genes, including 14 tRNA and 9 PCGs, and other 14 genes are coded by the light chain. The overall base composition of the genome is as follows: A (43.1%), T (29.4%), C (17.4%), G (10.2%), with an A + T content of 72.4%. All PCGs start with ATN codon. Four PCGs end with incomplete stop codon T, while nine PCGs stop with TAA and TAG. All traditional tRNA genes ranging from 60 to 70 bp have the canonical cloverleaf secondary structure, except for tRNA^Ser(AGN)^. The 16S and 12S rRNA genes of *T. obscurus* are 1280 bp and 780 bp in size, respectively. The A + T-rich regions show marked differences between Guiyang population (1177 bp) and Zunyi population (902 bp), which can be used as a powerful molecular marker for population investigation.

The ML and BI phylogenetic trees exhibited the same topology (Figure 1), which is largely similar to the reported molecular phylogenies (Zhang et al. 2016). Within the Cucujiformia, the Chrysomeloidea, Curculionoidea, and Tenebrionoidea formed a monophyletic lineage respectively. Within the Tenebrionoidea, the Tenebrionidae species are grouped together as a monophyletic lineage and then placed as the sister group of Mordellidae. In the family Tenebrionidae, *T. obscurus* was most closely related to *T. molitor*. Our study provides additional molecular data for subsequent studies on population structure, and management strategies for this important resource insect.

**Figure 1.**
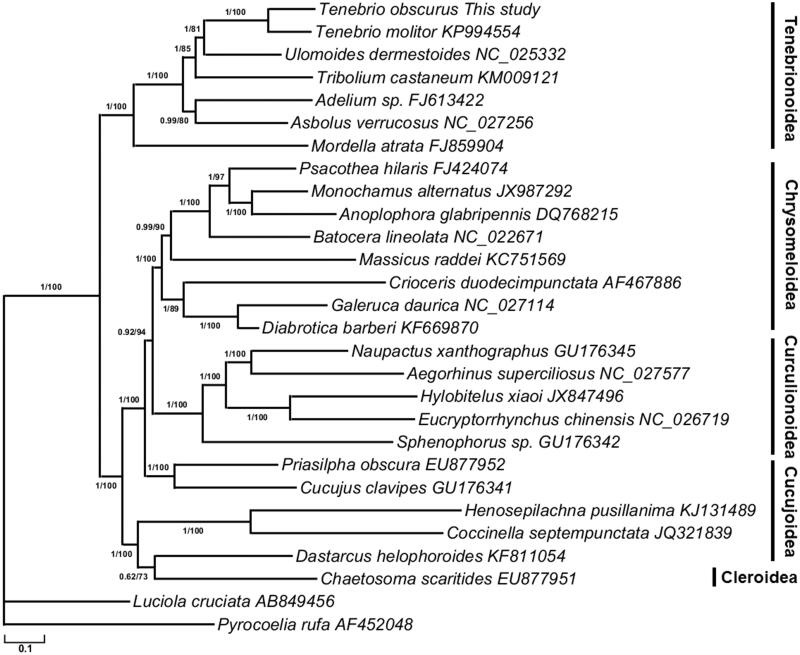
Phylogeny of 26 *Cucujiformia* species based on the maximum-likelihood (ML) and Bayesian inference (BI) analysis of the concatenated coding sequences of 13 mitochondrial PCGs. The nodes correspond to Bayesian posterior probabilities (left) and ML bootstrap support values in percentages (right, 1000 resamplings), respectively.
